# Evaluation of FABP1 as a biomarker of diabetic kidney disease in children with type 1 diabetes

**DOI:** 10.1007/s00431-026-07142-8

**Published:** 2026-06-16

**Authors:** Hadil Mohamed Aboelenin, Heba ElTaher, Rasha Elzehery, Sara Mahmoud Nosir, Mohammad Al-Haggar

**Affiliations:** 1 Department of Pediatrics, Endocrinology and Diabetes Unit, Faculty of Medicine, Mansoura University, Mansoura, Egypt; 2 Department of Pediatrics, Genetics Unit, Faculty of Medicine, Mansoura University, Mansoura, Egypt; 3 Department of Clinical Pathology, Faculty of Medicine, Mansoura University, Mansoura, Egypt; 4 Mansoura University Children Hospital, Mansoura, Egypt

**Keywords:** Fatty acid–binding protein, Type 1 diabetes mellitus, Diabetic kidney disease, Microalbuminuria

## Abstract

Diabetic kidney disease (DKD) is a chronic microvascular complication of diabetes mellitus and a leading cause of chronic kidney disease worldwide. Microalbuminuria remains the current gold standard for the diagnosis and staging of DKD; however, it often reflects established renal injury rather than early pathological changes. Fatty acid–binding protein 1 (FABP1), a cytoplasmic protein abundantly expressed in the renal proximal tubules, is released into the urine in response to tubular damage. FABP1 plays an essential role in intracellular fatty acid transport and metabolism and may serve as a potential biomarker for tubular involvement in pediatric diabetic kidney disease. A case–control study was performed on 90 children (30 T1DM with DKD, 30 T1DM without DKD [non-DKD], and 30 controls). Albumin–creatinine ratio (ACR) and serum FABP1 were ELISA measured. FABP1 was significantly higher in the DKD group versus non-DKD (*p* = 0.026) and controls (*p* = 0.009). FABP1 positively correlated with ACR (*r* = 0.25, *p* = 0.018), total cholesterol (*r* = 0.25, *p* = 0.019), and LDL-C (*r* = 0.25, *p* = 0.017). A cutoff > 189 ng/L discriminated DKD from non-DKD (AUC = 0.67, sensitivity 63%, specificity 73%).

*Conclusion*: Serum FABP1 may represent a complementary biomarker of tubular involvement in pediatric diabetic kidney disease.

**What is Known:**

• *Microalbuminuria detects DKD only after significant renal injury has occurred*.

• *FABP1 is released from proximal tubular cells in response to early tubular damage*.

**What is New:**

• *Serum FABP1 is associated with diabetic kidney disease in children with type 1 diabetes mellitus and correlates with albumin–creatinine ratio*.

• *FABP1 may provide additional information on tubular involvement when used alongside conventional glomerular markers*.

## Introduction

T1DM is a chronic disease featured by insulin deficiency secondary to pancreatic β cell loss with a subsequent development of hyperglycemia. It has micro- and macrovascular complications. Although good glycemic control has reduced the occurrence of these complications, many people with T1DM are still developing these complications [1].

DKD is a microvascular consequence of type 1 and type 2 diabetes that is poorly controlled and is defined by a progressive decline in GFR with ESRD, hypertension, and an increase in cardiovascular disease events [2]. DKD is divided into five stages; the first stage is hyperfiltration, hyperperfusion; the second stage is subclinical stage, increase in albumin excretion rate within normal range; the third stage is moderately increased albuminuria (microalbuminuria); the fourth stage is macroalbuminuria; and the last stage is ESRD [3].

According to KDIGO guidelines, albuminuria is categorized into three stages: A1: normal to mildly increased (< 30 mg/g creatinine), A2: moderately increased (30–299 mg/g creatinine)—formerly called microalbuminuria, and A3: severely increased (≥ 300 mg/g creatinine)—formerly called macroalbuminuria [4].

Microalbuminuria (A2 stage) screening is the gold standard diagnostic test for detection of DN in type 1 DM, and early detection is crucial for timely management [5]. From the age of 11 years and after 2 years of the development of diabetes, patients should have regular annual screening for microalbuminuria and the development of ESRD is predicted by persistent microalbuminuria [6]. However, there are some restrictions on the use of albuminuria as a biomarker for the diagnosis of DKD because not all diabetic children with proteinuria will exhibit a decline in kidney function, and there are other factors that can affect albuminuria levels and eGFR, including fever, infections, diet, stress, physical activity, hydration status, menstruation, and hyperglycemia [5].

As early progressive renal impairment (annual eGFR loss 3.3%) may occur before the onset of A2 stage and its progression to A3 stage, DKD can develop even in the absence of increased albuminuria [3].

In recent years, biomarkers reflecting tubular injury and interstitial pathology have gained increasing attention for the early detection of DKD. These biomarkers may provide additional diagnostic value and serve as complementary tools to albuminuria in identifying subclinical renal involvement. Candidate biomarkers have been selected based on experimental in vitro and in vivo evidence supporting their role in the pathophysiology of diabetic renal injury [7].

Importantly, early renal injury may be present even in normoalbuminuric patients, and urinary biomarkers have been proposed for earlier detection of subclinical diabetic kidney disease. This is supported by studies demonstrating elevated urinary VEGF-A, angiotensinogen, and transferrin in normoalbuminuric children with type 1 diabetes [8] as well as increased urinary inflammatory and tubular injury markers in early-stage nephropathy before the development of overt albuminuria [9]*.*

Fatty acid–binding protein 1 (FABP1) is a low-molecular-weight protein involved in intracellular fatty acid transport and lipid metabolism and is expressed in the proximal renal tubules. It has a distinct structural configuration compared with other members of the FABP family, allowing it to bind multiple ligands simultaneously [10, 11].

There are many conditions that lead to increase excretion of urine-derived FABP1 such as hyperglycemia, hypertension, proteinuria, and toxin-induced injury to the proximal renal tubule cells [12]. Urinary and serum FABP1 was shown to be good marker for detection of ESRD and ischemia damage caused by renal transplantation [13, 14].

Early identification of DKD is very important for early intervention, and detection of FABP1 in urine can be used as a diagnostic marker of early stages of kidney damage [15, 16].

Serum FABP1was proved as a novel biomarker of DKD in T2DM. However, data on FABP1 in pediatric T1DM are scarce [17].

This study investigated serum FABP1 as a potential biomarker for early detection of DKD in children with T1DM.

## Subjects and methods

This was a case–control study conducted between May 2022 and May 2023. The patients were recruited consecutively from the Outpatient Pediatric Clinic of Diabetes in Mansoura University Children’s Hospital (MUCH), Mansoura, Egypt. The study was conducted on 90 children: 60 children with T1DM from the Outpatient Clinic of Pediatric Diabetes and 30 age and sex-matched children as healthy control from the general pediatric clinic. The diabetic children were divided into two groups; the first group (G1) included patients with DKD, and the second group (G2) included patients without DKD. Exclusion criteria included patients with age more than 18 years, type 2 diabetes mellitus, associated kidney disease either congenital or acquired, those who had dehydration, those who received nephrotoxic drugs (e.g., aminoglycosides, NSAIDs, calcineurin inhibitors, chemotherapy, or recent contrast agents), as these agents may independently affect renal tubular function and FABP1 levels, patients with history of diabetic keto acidosis (DKA) during the previous 4 weeks, and presence of acute infection at the time of clinical and laboratory evaluation (e.g., febrile illness or clinically evident systemic or urinary infection).

- All patients were submitted to comprehensive history taking regarding age, sex and residence, age of onset of diabetes, duration of diabetes, total dose of insulin per kg received at the time of diagnosis, type of insulin regimen, and clinical examination.
- Weight, height, and body mass index (BMI) were evaluated by standard methods; their *Z* scores were evaluated based on Egyptian growth charts [18].
- Blood pressure was measured using a calibrated manual mercury sphygmomanometer with an appropriately sized cuff covering 80–100% of the arm circumference. Measurements were obtained in the seated position after at least 5 min of rest, and the average of two readings taken 2–3 min apart was recorded and the results then were plotted on blood pressure curves according to the age, sex, and height centiles.

### Laboratory investigations

Routine hematological and biochemical investigations were done including complete blood picture, fasting blood glucose, hemoglobin A1C, and albumin creatinine ratio (ACR). Serum creatinine-based eGFR (eGFR-Cr) was calculated using updated Schwartz formula [19]. Lipid profile (serum total cholesterol, triglycerides, LDL, and HDL) and HbA1c were measured by means of quantitative colorimetric ion exchange resin chromatography kits provided by Stan Bio Laboratory (Boerne, Texas). Calibrators referenced to National Glycohemoglobin Standardization Program (NGSP) and values of HbA1c were presented as the unit of a percentage (%). Serum creatinine (Cr, mg/dL) was measured on a Dimension Xpand Plus chemistry analyzer using its kits, which were supplied by Siemens Technology (Illinois). Serum total cholesterol and triglycerides were measured by colorimetric kit supplied by spinreact (Girona, Spain). Serum high-density lipoprotein (HDL) cholesterol was measured by a colorimetric kit supplied by Human Diagnostics (Wiesbaden, Germany). Low-density lipoprotein (LDL) cholesterol was estimated using the Friedewald formula: LDL (mg/dL) = [TC − HDL] − TG/5 L).

As regards plasma FABP1, the kit used a double-antibody sandwich enzyme-linked immunosorbent assay (ELISA) to measure the level of human fatty acid–binding protein 1 in samples.

Based on the results of urinary ACR, children with T1DM were divided into two groups: G2 without DKD (*n* = 30, UACR value < 30 mg/g creatinine) and G1 with DKD (*n* = 30, UACR value 30–299 mg/g creatinine).

### Ethical consent

The study protocol was approved by the Research Ethics Committee of Faculty of Medicine, Mansoura University (Code No. Ms/22.02.1859) and followed by the Institutional Research Board. Informed consent was obtained from the parents of the children participating in the study.

### Statistical analysis

Data were entered and analyzed by utilizing IBM-SPSS software (IBM Corp., Released 2020, IBM SPSS Statistics for Windows, Version 27, Armonk, NY). MedCalc® Statistical Software version 20 (MedCalc Software Ltd, Ostend, Belgium, https://www.medcalc.org; 2021) was used.

Qualitative data were initially tested for normality using Shapiro–Wilk’s test. Quantitative data were expressed as mean ± standard deviation if normally distributed or median and interquartile range (Q1 or 25th percentile–Q3 or 75th percentile) if not. The Mann–Whitney *U* test was utilized to compare nonnormally distributed quantitative data between two groups. The ANOVA test was utilized for comparison of normal distribution of quantitative data between more than two groups. The Kruskal–Wallis *H* test was utilized to compare nonnormally distributed quantitative data between at least two groups.

The receiver operating characteristic (ROC) curve assessment was utilized to find a cutoff value of a continuous variable that can discriminate between microalbuminuric (A2) and normoalbuminuric (A1) diabetic groups.

## Results

No significant differences were observed among the studied groups regarding demographic and clinical characteristics, including age, sex distribution, anthropometric measures, blood pressure, or diabetes-related variables (Table 1). Additionally, no significant differences were found between the two diabetic groups in terms of age at onset, duration of diabetes, insulin dose, HbA1c, or lipodystrophy.

**Table 1:** Clinical and demographic data of the three groups

Characteristic	DKD (*n* = 30)	T1DM without DKD (*n* = 30)	Controls (*n* = 30)	*p* value
Sex (M/F), *n* (%)	13/17 (43.3/56.7)	17/13 (56.7/43.3)	18/12 (60/40)	0.392
Age (years)^†^	13.3 (13–15)	13 (12–15)	13 (12–14)	0.477
Age at onset (years)^†^	7.5 (6–9)	8 (5.75–10)	—	0.547
Duration of T1DM (years)^†^	6 (4.88–7)	6 (4.38–7.25)	—	0.687
Insulin TDD (U/kg/day)^†^	1.0 (0.80–1.18)	1.0 (0.80–1.33)	—	0.688
HbA1c (%)^†^	8.9 (8.17–10.0)	9.5 (8.28–10.23)	—	0.510
Lipodystrophy, *n* (%)	4 (13.3)	3 (10.0)	—	1.000
SBP (mmHg)^†^	115 (110–120)	117.5 (110–120)	110 (110–120)	0.486
DBP (mmHg)^†^	70 (70–80)	70 (70–80)	80 (70–80)	0.364
BMI (kg/m^2^)^‡^	24.7 ± 4.0	23.5 ± 3.1	24.0 ± 3.0	0.465

^†^Median (interquartile range)

^‡^Mean ± standard deviation

Regarding laboratory findings (Table 2), both diabetic groups showed significantly higher total cholesterol and LDL-C levels compared to controls, with significantly higher levels observed in the DKD group compared to the non-DKD group. No significant differences were detected in triglycerides, HDL-C, TG/HDL-C ratio, eGFR, serum creatinine, or HbA1c among the groups. Although TG/HDL-C ratio was higher in the DKD group, this difference did not reach statistical significance.

**Table 2:** Laboratory data of examined groups

Parameter	DKD (*n* = 30)	T1DM without DKD (*n* = 30)	Controls (*n* = 30)	*p* value
Triglycerides (mg/dL)	90 (72–104)	80 (66–113)	81 (66–113)	0.520
Total cholesterol (mg/dL)	180 (150–210)	164 (147–187)	124 (111–148)	< 0.001
HDL-C (mg/dL)	41.9 (38.9–44.8)	41.9 (38.8–47.3)	41.0 (37.0–49.2)	0.992
LDL-C (mg/dL)	125.5 (92.8–147.0)	105.5 (83.0–119.5)	60.5 (51.0–70.8)	< 0.001
TG/HDL-C ratio	2.25 (1.57–2.65)	1.95 (1.51–2.64)	2.0 (1.74–2.15)	0.569
eGFR (mL/min/1.73 m^2^)	94 (89–106.25)	96 (89.8–112.5)	99.5 (95.5–110.5)	0.073
ACR (mg/g)	70 (59–100)	14.1 (10.38–17.25)	15.5 (12–18.25)	< 0.001
Serum creatinine (mg/dL)	0.7 (0.6–0.7)	0.7 (0.6–0.7)	0.6 (0.6–0.7)	0.146
HbA1c (%)	8.9 (8.17–10.0)	9.5 (8.28–10.23)	—	0.510
FBG (mg/dL)	230 (215–251)	210 (199–229)	—	0.018
FABP1 (ng/L)	213.5 ± 69.2	176.0 ± 44.4	170.7 ± 47.8	0.006

The DKD group demonstrated significantly higher ACR and fasting blood glucose levels compared to the non-DKD group. Serum FABP1 levels were significantly elevated in the DKD group compared to both the non-DKD and control groups, while no significant difference was observed between the latter two groups.

Correlation analysis (Table 3) revealed a significant positive correlation between FABP1 and ACR, total cholesterol, and LDL-C, whereas no significant correlations were found with other clinical or laboratory parameters.

**Table 3:** Significant correlations between serum FABP1 and selected clinical and laboratory characteristics

Category	Parameter	Spearman’s *r*	*p* value
Renal markers	Albumin–creatinine ratio (ACR)	0.249	**0.018**
	eGFR	− 0.109	0.308
	Serum creatinine	0.090	0.397
Glycemic profile	HbA1c	0.238	0.067
	Fasting blood glucose (FBG)	0.191	0.143
	Duration of DM	0.057	0.668
	Age at onset of DM	0.139	0.288
	Total insulin dose	0.035	0.791
Lipid profile	Total cholesterol	0.246	**0.019**
	LDL-cholesterol	0.252	**0.017**
	HDL-cholesterol	− 0.186	0.079
	Triglycerides	− 0.010	0.927
Anthropometric measures	Age	0.044	0.677
	Weight	0.138	0.194
	Weight *Z* score	0.114	0.284
	Height	0.113	0.290
	Height *Z* score	0.115	0.281
	BMI	0.009	0.937
	BMI *Z* score	0.035	0.745
	Waist circumference	0.109	0.306
	Hip circumference	0.162	0.128
	Waist/hip ratio	− 0.073	0.496
Blood pressure	Systolic BP	− 0.032	0.768
	Diastolic BP	− 0.169	0.111
Hematological parameters	Hemoglobin	0.160	0.132
	WBC count	− 0.041	0.701
	Platelet count	0.012	0.912

Values in bold indicate *P* value significance

**Fig. 1:**
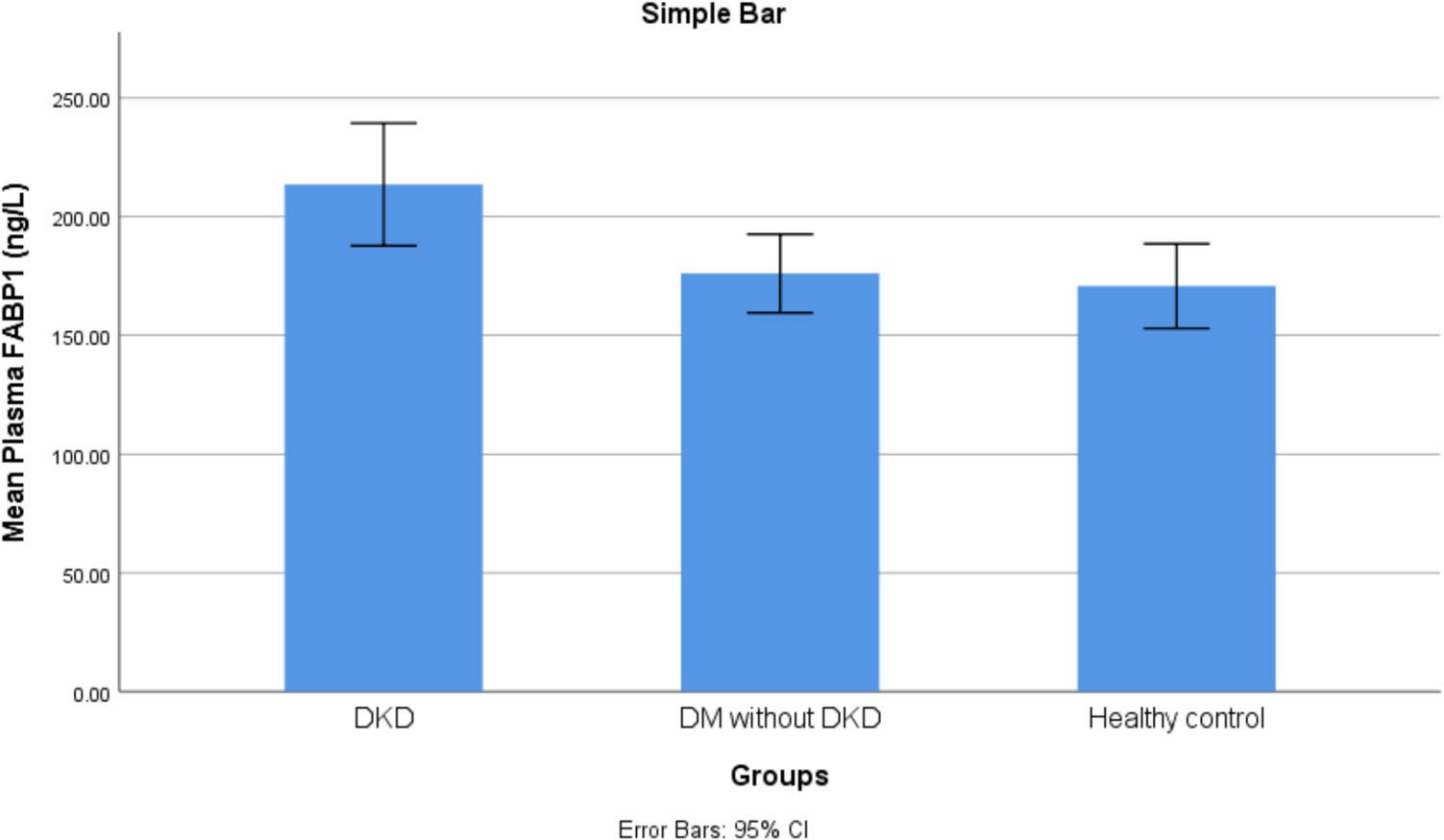
Comparison of plasma FABP1 between the three study groups

**Fig. 2:**
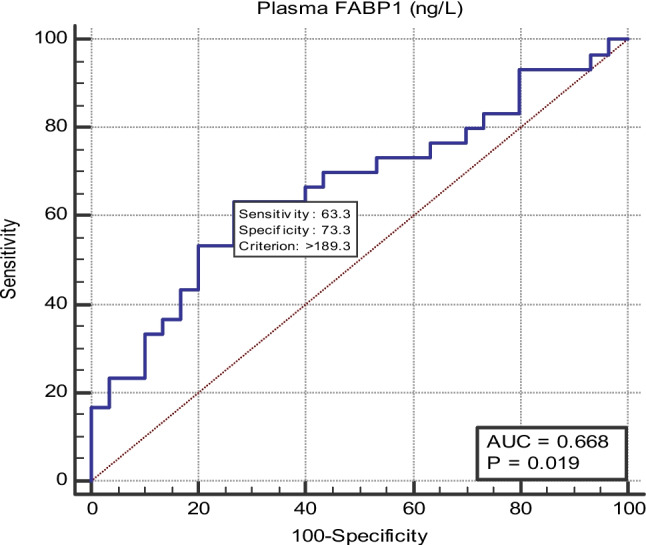
ROC curve for FABP1 in discriminating DKD group (G1) vs. DM without DKD group (G2)

ROC curve analysis (Table 4, Figs. 1 and 2) show that FABP1 at cutoff values of > 189.3 and > 225.2 is a statistically significant discriminator of DKD group vs. DM without DKD and control groups, respectively. However, FABP1 was not able to discriminate DM without DKD group vs. control group.

**Table 4:** Cutoff values of FABP1 for discriminating the three groups

Groups	Cutoff	AUC (95% CI)	*p* value	Sensitivity	Specificity
DKD vs. DM without DKD	> 189.3	0.668 (0.534–0.784)	**0.019**	63.3%	73.3%
DKD vs. control	> 225.2	0.677 (0.543–0.792)	**0.011**	43.3%	86.7%
DM without DKD vs. control	> 137.97	0.513 (0.381–0.645)	0.863	80%	36.7%

Values in bold indicate *P* value significance

## Discussion

Diabetic kidney disease is the most severe diabetes complication as it leads to ESRD and high risk of death [20]. FABP1 linked to DKD has been suggested to be a predictor for progression to microalbuminuria (A2) in patients with type 1 diabetes; whether FABP1 would be a more sensitive marker of DKD than albumin excretion rate is not well understood [21].

Regarding FABP1, it was statistically significantly higher in the DKD group (G1) versus DM without DKD (G2) and control (G3) but not higher in G2 in comparison to G3 and this aligns with Abo El-Asrar et al. [22] who found that FABP1 levels were significantly higher among type 1 diabetic patient with and without nephropathy compared with healthy controls with the highest levels among patients with nephropathy. Moreover, Tsai et al. [10] demonstrated that plasma FABP1 levels were significantly associated with diabetic nephropathy in type 2 DM.

Numerous studies have identified lipotoxicity as a significant factor in the progression of DKD [23].

We observed a statistically significant positive correlation between FABP1 and ACR, total cholesterol, and LDL-C but not with any of the other studied parameters. These findings are partly consistent with previous research. Tsai et al. [10] reported that plasma FABP1 levels increased with the severity of DN and showed positive associations with total cholesterol and LDL-C among patients with diabetes [1]. Similarly, Hassaan et al. [24] found significant positive correlations between FABP1 and albumin–creatinine ratio as well as serum creatinine and urea and a negative correlation with eGFR. In contrast, Abo El-Asrar et al. [22] reported that FABP1 correlated not only with total cholesterol but also with systolic blood pressure, fasting blood glucose, and HbA1c among patients with type 1 diabetes mellitus. In our study, none of the participants had a reduced GFR; however, prior studies consistently demonstrated an inverse relationship between FABP1 and eGFR.

In the present study, FABP1 showed a statistically significant ability to discriminate between patients with DKD G1 and both diabetic patients without DKD and healthy controls (G2 and G3). The optimal cutoff values of FABP1 were > 189.3 ng/mL and > 225.2 ng/mL, yielding sensitivity, specificity, and AUC values of 63.3%, 73.3%, and 0.668 and 43.3%, 86.7%, and 0.667, respectively. Conversely, FABP1 failed to distinguish diabetic patients without DKD (G2) from control(G3) (sensitivity 80%, specificity 36.7%, and AUC 0.513). These results are consistent with Suzuki et al. [25] who conducted his study on 356 type 2 diabetic patients divided according to the degree of albuminuria and he found a significant association between the stage of diabetic nephropathy and FABP-1 [21].

The ROC curves of FABP1 concentrations in Tsai et al. [10] showed that the ROC curve to detect DKD demonstrated an area under the curve (AUC) of 0.780 for FABP1. A plasma FABP1 concentration of > 33.8 ng/mL was accompanied by DKD, with a sensitivity of 75% and specificity of 75.5%. On the other hand, the ROC curve analysis in Panduru et al.’s [26] study did not reveal benefits of utilizing L-FABP for prediction of the progression. The ROC curve analysis of Abo El-Asrar et al. [22] showed that FABP1 could differentiate patients with and without nephropathy with balanced sensitivity and specificity; this suggests that monitoring of serum FABP1 in addition to urine screening for albuminuria may help in the diagnosis of renal impairment in pediatric patients with T1DM before a significant rise in serum creatinine.

The lack of significant FABP1 elevation in normoalbuminuric (A1) diabetic children suggests that FABP1 may not detect the earliest preclinical renal changes but rather reflects tubular involvement once diabetic kidney disease becomes biochemically evident.

Regarding the lab data, we found statistically significant difference in serum total cholesterol, LDL-C, and ACR, between the three study groups. We observed that the serum total cholesterol and LDL-C were statistically significantly higher in the two diabetic groups vs. the healthy control group; also, Abo El-Asrar et al. [22] found that total cholesterol was significantly higher in type 1 diabetic patients with early nephropathy than those without nephropathy and Palazhy and Viswanathan [27] showed that TC, TG, and LDL-C levels were significantly higher among the nephropathy patients indicating poor control of DM affects lipid profile and leads to diabetic kidney disease. Regarding TG/HDL-C ratio, we found that it was higher in our study in diabetic nephropathy vs. the two other groups but this difference was not statistically significant. Abo El-Asrar et al. [22] found that triglycerides were significantly higher in type 1 diabetic patients with early nephropathy than those without nephropathy. This difference might be due to the difference in duration of the disease and stage of DKD between the two studies.

As regards serum creatinine and e GFR, we did not observe a significant difference between the three groups; Chae et al. [28] also showed no significant difference between the normoalbuminuric (A1) group and the microalbuminuric (A2) group (*p* = 0.066), and none of our cases developed stage 1 V DKD. Regarding HbA1c, our study showed no statistically significant difference between the two groups of diabetes (*p* = 0.0510), but Elley et al. [29] reported presence of positive correlation between progression to diabetic nephropathy and HB A1C. Also Chae et al. [28] showed that HbA1c level was significantly higher in the DKD group than in the diabetic non-KD group (*p* = 0.037). We observed that FBG was statistically significantly higher in the diabetic KD group vs. the diabetic non-KD group. In agreement with our results, Abo El-Asrar et al. [22] found FBG was also significantly higher in type 1 diabetic patients with early nephropathy than those without nephropathy; this may be attributed to the effect of hyperglycemia on the renin–angiotensin–aldosterone system (RAAS). Miller [30] demonstrated that hyperfiltration responses to clamped hyperglycemia are related to intrarenal RAAS activation.

Regarding clinical and demographic data, there were no statistically significant differences between the three groups regarding age and sex, and similar to our findings, other investigations disputed the existence of gender differences between diabetic and nondiabetic KD patients, claiming that no distinction between men and women was discovered [31].

The three study groups showed no significant difference between the three study groups regarding weight *Z* score, height *Z* score, and BMI *Z* score but Maric-Bilkan [32] found that obesity is a major risk factor to develop DKD, Kordonouri [33] found that short stature among patients with T1DM is associated with increased risk of developing nephropathy, and Katz et al. [34] found that the rise in BMI *Z*-score is associated with developing DN in patients with mean 10 years duration of diabetes and mean age 21 years.

As regards waist/hip ratio, our study showed no significant difference between the three groups (*p* = 0.376), as regards waist/hip ratio (*p* = 0.121), similar to the study of Tsai et al. [10] who revealed no significant difference in waist/hip ratio between diabetic KD and non-KD groups.

We did not observe a significant difference between the three study groups regarding systolic blood pressure (*p* = 0.486), but Abo El-Asrar et al. [22] found that the disease duration and systolic blood pressure were significantly higher in DKD. This difference may be due to the shorter duration of diabetes in our case study.

## Conclusion

Serum FABP1 may represent a supplementary biomarker reflecting early tubular injury in children with type 1 diabetes mellitus. However, due to its moderate diagnostic accuracy, it should be considered complementary to albumin–creatinine ratio rather than a superior alternative. Further large-scale prospective studies are required to confirm its diagnostic and prognostic value.

## Study limitation and future directions

Small simple size and single-center study limit generalizability, and therefore, future multicenter longitudinal research studies have to be contemplated to evaluate FABP1 alongside other tubular markers to establish reference ranges and clinical cutoff values for pediatric DKD.

## Data Availability

No datasets were generated or analysed during the current study.

## Abbreviations

ACR: Albumin to creatinine ratio
BMI: Body mass index
DKA: Diabetic ketoacidosis
DM: Diabetes mellitus
DKD: Diabetic kidney disease
DBP: Diastolic blood pressure
eGFR: Estimated glomerular filtration rate
ESRD: End-stage renal disease
FABP1: Fatty acid–binding protein 1
HDL: High-density lipoprotein
HDL-C: High-density lipoprotein-cholesterol
KDIGO: Kidney Disease: Improving Global Outcomes
LDL: Low-density lipoprotein
SBP: Systolic blood pressure
TDD: Total daily dose
WHR: Waist/hip ratio
